# Cartilage repair strategies in the knee according to Dutch Orthopedic Surgeons: a survey study

**DOI:** 10.1007/s00402-023-04800-6

**Published:** 2023-02-21

**Authors:** R. M. Jeuken, P. P. W. van Hugten, A. K. Roth, T. A. E. J. Boymans, J. Caron, A. Weber, R. J. H. Custers, P. J. Emans

**Affiliations:** 1 Department of Orthopedic Surgery, Joint Preservation Clinic, Caphri School for Public Health and Primary Care, Maastricht University Medical Center, Maastricht, The Netherlands; 2 Department of Orthopedic Surgery, St Elisabeth Ziekenhuis, Tilburg, The Netherlands; 3 Department of Orthopedic Surgery, University Medical Center Utrecht, Utrecht, The Netherlands

**Keywords:** Cartilage, Repair, Knee, Survey, Dutch

## Abstract

**Background:**

This study surveyed Dutch orthopedic surgeons on the management of cartilage defects in the knee and the adherence to the recently updated Dutch knee cartilage repair consensus statement (DCS).

**Methods:**

A web-based survey was sent to 192 Dutch knee specialists.

**Results:**

The response rate was 60%. Microfracture, debridement and osteochondral autografts are performed by the majority, 93%, 70% and 27% of respondents, respectively. Complex techniques are used by < 7%. Microfracture is mainly considered in defects 1–2 cm^2^ (by > 80%) but also in 2–3 cm^2^ (by > 40%). Concomitant procedures, e.g., malalignment corrections, are performed by 89%. Twenty-one percent of surgeons treat patients aged 40–60 years. Microfracture, debridement and autologous chondrocyte implantation are not considered to be highly affected by age > 40 years by any of the respondents (0–3%). Moreover, for the middle-aged there is a large spread in treatments considered. In case of loose bodies, the majority (84%) only performs refixation in the presence of attached bone.

**Conclusion:**

Small cartilage defects in ideal patients may be well treated by general orthopedic surgeons. The matter becomes complicated in older patients, or in case of larger defects or malalignment. The current study reveals some knowledge gaps for these more complex patients. Referral to tertiary centers might be indicated, as is stated by the DCS, and this centralization should enhance knee joint preservation. Since the data from present study are subjective, registration of all separate cartilage repair cases should fuel objective analysis of clinical practice and adherence to the DCS in the future.

## Introduction

Articular cartilage defects in the knee occur frequently and may cause considerable pain and disability [1–3]. Cartilage regeneration or repair techniques may be indicated when cartilage defects become symptomatic.

Current techniques used in clinical practice include marrow stimulating repair techniques such as microfracture (MF) and its augmentations, regenerative techniques such as autologous chondrocyte implantation (ACI) and regenerative osteochondral scaffolds, and bone-based repair techniques—i.e., depending on osseointegration—such as osteochondral grafting using autografts or allograft transplantations (OAT and OCA) and focal knee resurfacing implants (FKRIs) [4]. MF augmentations include interventions such as autologous matrix-induced chondrogenesis. Regenerative osteochondral scaffolds include treatments such as Trufit™ (Smith and Nephew), MaioRegen (Finceramica) amd Agili-C™ (CartiHeal). Non-degradable bone-based FKRIs include HemiCAP^®^ (Arthrosurface), Episealer^®^ (Episurf) and BioPoly^®^ RS Femoral Condyle (BioPoly).

In an attempt to provide guidance within the complex field of cartilage regeneration and repair, several international cartilage experts have composed guidelines or ‘treatment algorithms’. The Dutch Orthopedic Society (Nederlandse Orthopedie Vereniging—NOV) cartilage repair consensus statement for (osteo)chondral surgical repair (abbreviated Dutch Consensus Statement; DCS) was first published in 2011 and were updated in 2019 (Table 1) [5]. Although the Netherlands is known for its excellent healthcare quality and registration [6], there is no separate registration of cartilage repair, i.e., there are no cartilage specific procedural terminology (CPT) billing codes. There is consequently no information about the perception and adherence to the DCS.

**Table 1:**
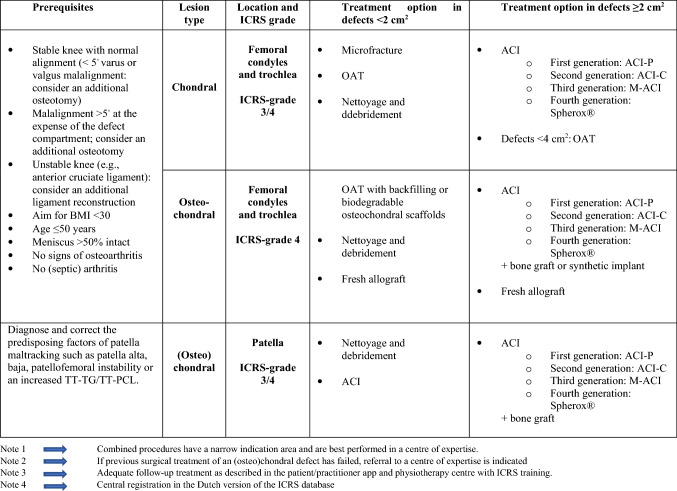
The 2019 Dutch Consensus Statement concerning cartilage defect repair in the knee

*ICRS* International Cartilage Repair Society, *OAT* Osteochondral Autologous Transplantation, *ACI* Autologous Chondrocyte Implantation, *BMI* Body Mass Index, *TT-TG* tibial-tuberosity to trochlear groove distance, *TT-PCL* tubercle-posterior cruciate ligament

The objective of this survey study was to provide insight in the applied cartilage repair techniques and adherence to the DCS in the Netherlands. In addition, this study emphasized the patient age-related considerations by orthopedic surgeons. With relatively little literature available related to the treatment of the middle-aged population, insights by orthopedic surgeons concerning their patient age-related considerations in cartilage repair aid in understanding this knowledge gap [3].

## Methods

### Participants

The survey recipients consisted of members of the Dutch Knee Society (DKS), which is part of the Dutch Orthopedic Society (Nederlandse Orthopeden Vereniging—NOV) and the Dutch Association for Arthroscopy (Nederlandse Vereniging voor Arthroscopie—NVA), totaling 192 orthopedic knee surgeons.

### Questions

Three specialized cartilage orthopedic surgeons (JC, RC, and PE) and two residents in training (RJ and PvW) prepared questions for this survey. Questions were critically analyzed during one general meeting and three digital meetings until consensus was reached. The survey consisted of a total of 19 questions related to the treatment of cartilage defects. Questions were written in Dutch. For the purpose of the current international publication, answers were translated into English by a native English-speaking author (AW), as shown in Appendix 1.

The survey consisted of 12 general questions, including questions related to the surgeon’s experience, characteristics of typically treated patients, defect type, utilization of available therapies, and application of concomitant treatments. The general questions were followed by seven in-depth questions related to the treatment choice for different defect characteristics using the International Cartilage Repair and Joint Preservation Society (ICRS) scoring, the strategy for patients in different age categories and treatment preference for rare defects and loose cartilage bodies. In addition, a qualitative assessment was performed to assess specific rehabilitation protocols. The adherence of orthopedic surgeons to existing guidelines was evaluated using the general and in-depth questions such as cut-off points for age and body mass index (BMI), treatment choice for a given size and depth of defect, the indication and application of additional surgical techniques, and the utilization of rehabilitation protocols.

### Survey distribution

The web-based survey was created in SurveyMonkey^®^ (San Mateo, CA, USA). Orthopedic surgeons were invited by e-mail to participate in the survey. To increase the response rate, two subsequent follow-up e-mails were sent after 3 and 6 weeks. Using IP-based duplicate protection, orthopedic surgeons were prevented from completing the questionnaire twice. This study was performed according to Best Practices for Survey Research Reports [7].

## Results

### Participants and general questions

The response rate was 60% and 75% of respondents (*n* = 115) completed the survey, resulting in an overall completion rate of 44%. Respondent demographics are shown in Table 2.

**Table 2:**
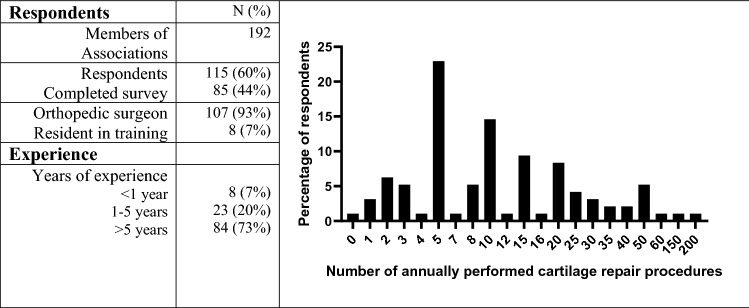
Characteristics and experience of respondents to the national cartilage repair survey

Note in the histogram that the numbers on the *X*-axis are not linear

*SD* standard deviation

**Fig. 1:**
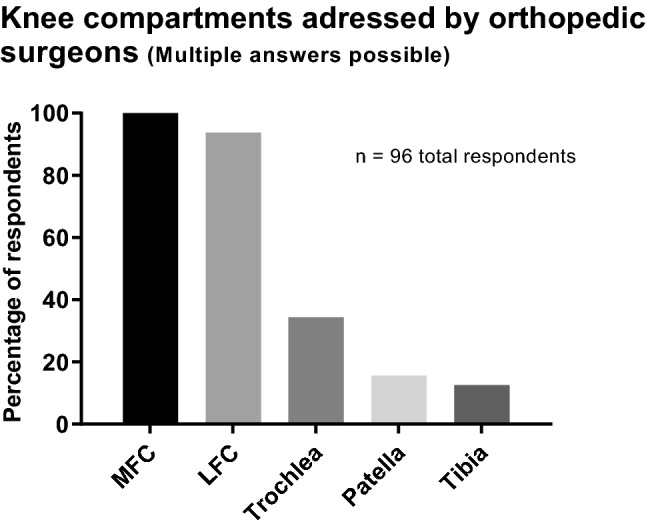
Typical knee compartments addressed according to respondents. The question leading up to these results was ‘I apply cartilage repair to the following compartments of the knee:’ *MFC* medial femoral condyle, *LFC* lateral femoral condyle

**Fig. 2:**
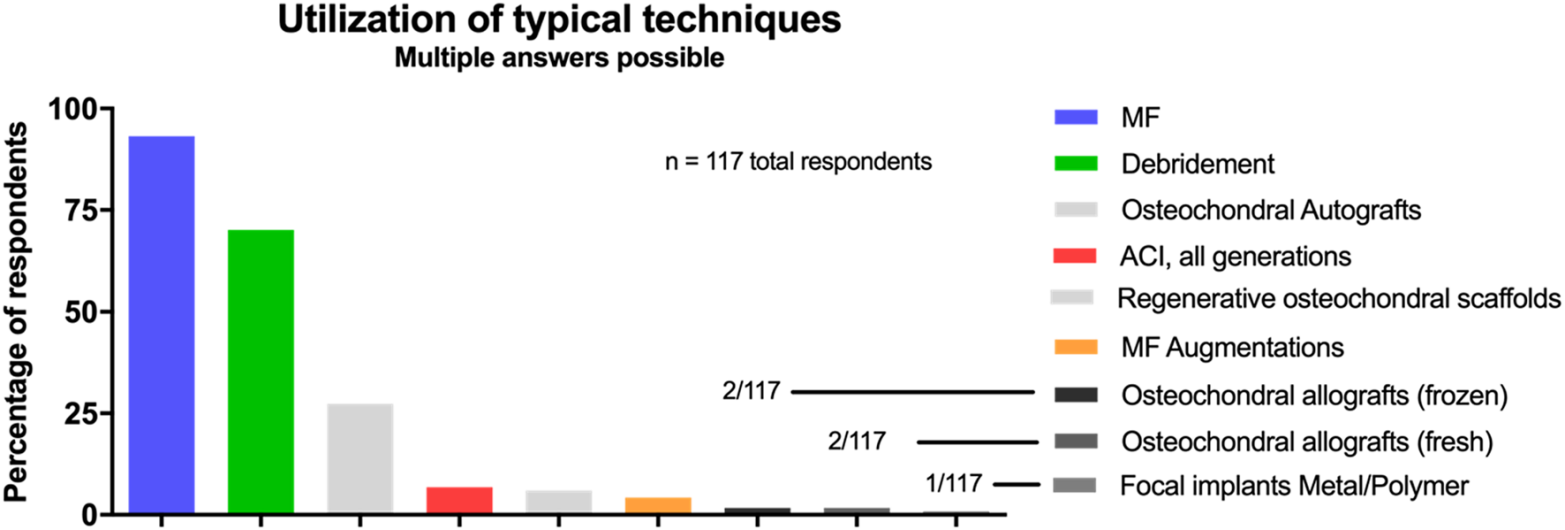
Utilization of typical cartilage repair techniques. The question leading up to these results was **A**: ‘I use the following techniques for cartilage repair of symptomatic cartilage defects’. *MF* microfracture, *ACI* autologous chondrocyte implantation; Regenerative osteochondral scaffolds include treatments such as Trufit™ (Smith and Nephew), MaioRegen (Finceramica), Agili-C™ (CartiHeal); MF Augmentations: MF augmentations such as autologous matrix-induced chondrogenesis. Focal implants metal/polymer includes treatments such as HemiCAP^®^ (Arthrosurface), Episealer^®^ (Episurf) and BioPoly^®^ RS Femoral Condyle (BioPoly)

Ninety-nine per cent of the respondents perform cartilage repair on the medial femoral condyle, 93% on the lateral femoral condyle, 34% on the trochlea, 16% on the patella and 13% on the tibia plateau, see Fig. 1. When asked which surgical techniques surgeons utilize, 95% of the respondents indicated they use MF, 71% use debridement, 2% use osteochondral autografts, 6% use ACI and degradable FKRIs, 6% use MF augmentations, 2% use fresh frozen osteochondral allografts and 1% use non-degradable FKRIs, see Fig. 2.

**Fig. 3:**
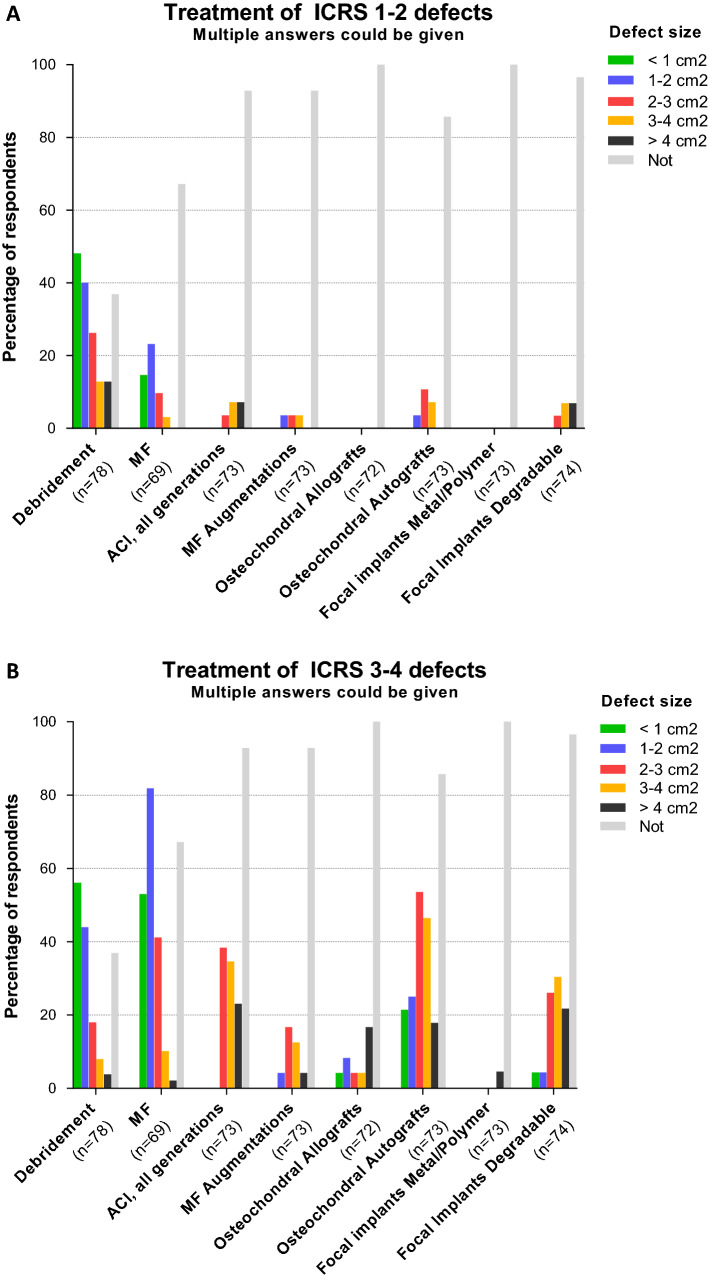
Use of different cartilage defect repair techniques for defects with ICRS 1/2 or 3/4 depths and different sizes. The question leading up to these results was **A:** ‘I would treat symptomatic, ICRS grade 1/2, cartilage defects with a maximum size of, with the following techniques’; and **B**: ‘I would treat symptomatic, ICRS grade 3/4, cartilage defects with a maximum size of, with the following techniques’. *MF* microfracture, *ACI* autologous chondrocyte implantation; Regenerative osteochondral scaffolds include treatments such as Trufit™ (Smith and Nephew), MaioRegen (Finceramica), Agili-C™ (CartiHeal); MF Augmentations: MF augmentations such as autologous matrix-induced chondrogenesis. Focal implants Metal/Polymer includes treatments such as HemiCAP^®^ (Arthrosurface), Episealer^®^ (Episurf) and BioPoly^®^ RS Femoral Condyle (BioPoly)

**Fig. 4:**
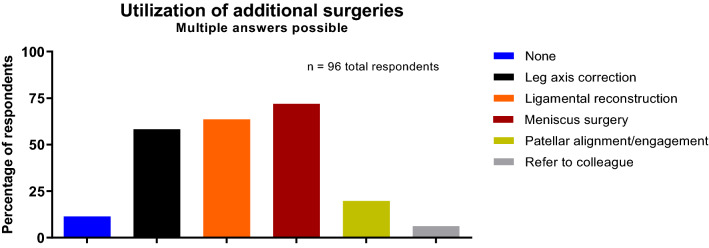
Utilization of additional surgeries. The question leading up to these results was ‘In patients with a symptomatic cartilage defect I apply the following techniques in addition to cartilage repair:’. Ligamental reconstruction includes cruciate and or collateral ligaments; Meniscus surgery includes all meniscus surgeries such as suturing, regenerative procedures, allografts and biomaterial implants

When faced with ICRS grade I/II defects, most respondents opt for debridement regardless of the defect size, see Fig. 3a. For ICRS grade III/IV defects up to 3 cm^2^, debridement and MF are both popular techniques, while the DCS maintains 2 cm^2^ as upper limit. Most surgeons indicate that they treat defects of 3–4 cm^2^ using osteochondral autografts and defects larger than 4 cm^2^ with ACI, see Fig. 3b. When indicated, 89% of surgeons perform concomitant surgeries; 70% perform meniscal augmentations, 64% perform ligamental reconstructions, 57% perform correction of the leg axis and six per cent answered other, see Fig. 4.

An upper BMI (kg/m^2^) limit of 30 has been adopted by 46% of the respondents in accordance with the DCS. BMI was set at unlimited by 24%, at 35 by 21%, at 40 by 5% and at 25 by 4%. Seventy-two per cent of the respondents treat patient who smoke and 28% indicated they do not treat smoking patients.

**Fig. 5:**
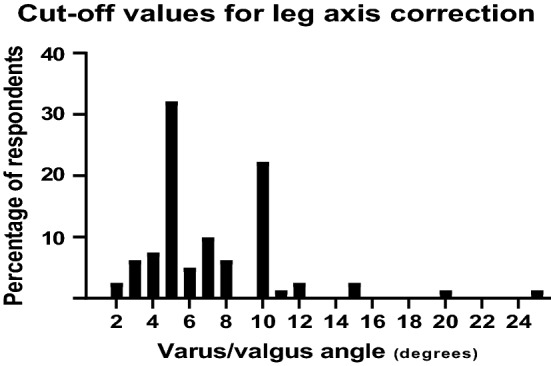
Histogram with the cut-off values of varus/valgus angle (degrees) indicated for leg-axis correction. The question leading up to these results was ‘Starting from how many degrees (varus/valgus) would you carry out a surgical correction of the leg axis? (open question)’

The median angle at which the respondents performed leg axis correction is 6°, the mode 5°, of which the latter is in accordance with the DCS, see Fig. 5. The majority (57%) of respondents prescribes a specific rehabilitation protocol for cartilage repair with varying strategies, but none of them mentioned specifically to employ an ICRS trained physiotherapist as dictated by the DCS, see Table 3. When indicated, 95% of respondents indicated to refer to a tertiary center.

**Table 3:** The question leading up to these results was: ‘My clinic has a specific cartilage repair rehabilitation protocol’

Respondents having a specific rehabilitation protocol and its qualitative assessment	
No	Yes
43%	57%
	‘Six weeks 50% load bearing, frequent cycling on home trainer’
	‘Dependent on defect location’
	‘Progressive loading’
	‘Six weeks unloaded, then 6 weeks unloader brace’
	‘Physiotherapy’
	‘I adapt my rehabilitation protocol to defect location and size and patient specifics’
	‘Brace’
	‘For MF on the medial or lateral femoral condyle: 6 weeks onloaded, then progressive loading up to three months. For MF in the patellofemoral joint, I consider a brace for range of motion restriction’
	‘Phased’
	‘Six weeks 10% loading’
	‘Six weeks permissive weightbearing under supervision of a physiotherapy’
	‘Six weeks 50% weightbearing, also depending on defect location, maximum flexion 90°’
	‘For MF 6 weeks unloading’
	‘Physiotherapy’
	‘Depending on defect location. On loaded parts of the femoral condyle 50% loading during the first six weeks’
	‘No brace, 6 weeks partially loading with a physiotherapist, 4 months no peak or pivoting movements’
	‘Two weeks brace and passive range of motion, 6 weeks onloaded’
	‘Onloaded’
	‘Cyclic exercises, 6 weeks unload, then progressive loading’
	‘Six weeks unload, then 6 weeks progressive loading supervised by physiotherapist. Refrain from loaded roll/glide movements for three months. Playing sports after six weeks’
	‘Six weeks crutches: 4 weeks unloaded, then progressive loading’
	‘Using an app’
	‘Unloaded/partially loaded for 6 weeks without restrictions in range of motion. Then functional loading. Sports only after 12 months’
	‘MF protocol’
	‘Depending on defect location’
	‘Unloaded, physiotherapy, brace depending on location’
	‘Six weeks hinge brace, partially loaded, then progressive loading’
	‘Six weeks unloaded, then 6 weeks progressive loading’
	‘Six weeks onloaded’
	‘Condyle protocol, patellofemoral protocol, combined protocol’
	‘Six weeks unloaded and 90° range of motion restriction’
	‘Partially loading and sometimes corrective brace’
	‘Six weeks unloaded, crutches, physiotherapy’
	‘Six weeks unloaded, restriction range of motion depending on location’
	‘Six weeks 50% loading and then progressive loading’

Respondents were asked to answer yes or no and elaborate on the protocol if they answered yes

### In-depth questions

**Fig. 6:**
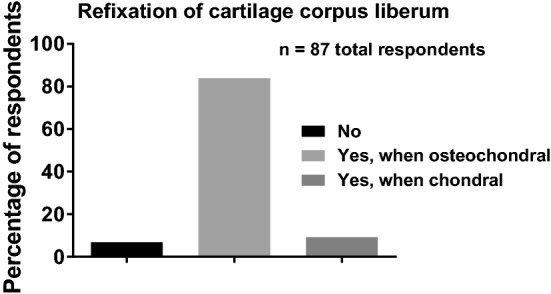
Refixation strategies when encountering a loose cartilage body, i.e., corpus liberum. The question leading up to these results was ‘In case of a cartilage corpus liberum I attempt refixation:’

When the respondents were asked if they would fixate a loose body, 84% responded that they fixate an osteochondral loose body, 9% fixate a chondral loose body and 7% would not attempt any fixation, see Fig. 6. Twelve per cent of the respondents treat ICRS grade 5 lesions (deeper than 6.5 mm). Eighty-four per cent of the respondents treats single lesions, 35% treats multiple lesions, and 8% treats kissing lesions. Sub-analysis revealed that 75% of respondents who work in a tertiary cartilage expert clinic would treat multiple and kissing lesions, whereas 28% of surgeons in a non-expert clinic would do this.

#### Patient age-related considerations

**Fig. 7:**
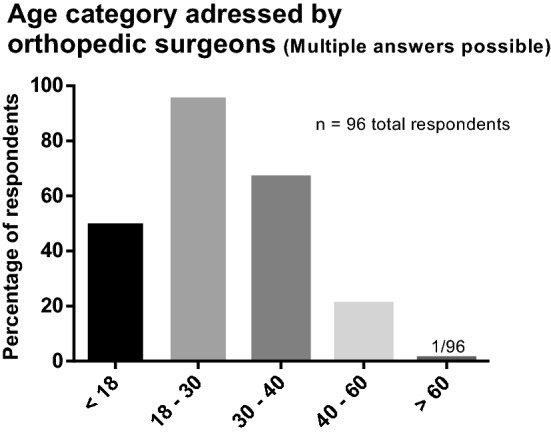
Typical age of patients on which cartilage repair is performed by the respondents. The question leading up to these results was ‘In general I treat cartilage defects within the following age categories:’

The vast majority of the respondents (96%) treat patients aged 18–30 years and 67% treats patients 30–40 years. Fifty per cent treats patients under the age of 18 years, 21% treats patients 40–60 years, and one per cent treats patients older than 60 years, see Fig. 7.

**Fig. 8:**
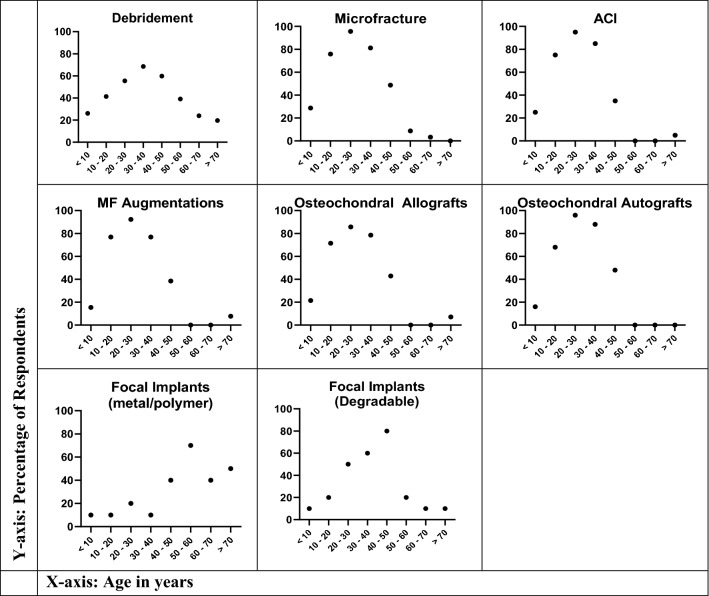
Techniques that were considered by the respondents for different patients’ age. The question leading up to these results was ‘I would consider the following techniques for the following age categories, irrespective of ICRS grade:’ *ACI* autologous chondrocyte implantation; MF augmentations: for example autologous matrix-induced chrondrogenesis; Focal Implants (Metal/Polymer): focal implants such as HemiCAP^®^ (Arthrosurface), Episealer^®^ (Episurf) and BioPoly^®^ RS Femoral Condyle (BioPoly); Focal implants (Degradable): Regenerative osteochondral scaffolds include treatments such as Trufit™ (Smith and Nephew), MaioRegen (Finceramica), Agili-C™ (CartiHeal)

When stratifying the different treatments by age group, most surgeons indicated to consider microfracture, debridement, MF augmentations, osteochondral autografts and allografts, ACI, in the categories ≤ 10 up to 40 years of age. The ultimate treatment choice thus appears to be dictated by defect characteristics in accordance with the treatment algorithms for this age group. For the age groups 40–50 and 50–60 years, the degree of agreement between respondents appears to decline, see Fig. 8. On a 5-point scale, debridement and MF augmentations are considered (58% of respondents) least affected by being middle-aged (40–65). Common treatments (debridement, MF) are not considered highly affected by middle age by any of the respondents (0–3% of respondents). Nine per cent of respondents considered allografts and biodegradable osteochondral scaffolds to be highly affected by age.

## Discussion

This was the first national survey on cartilage repair in the Netherlands. The response rate of 60% was considered to be adequate, given the typical response rate of 37–51% for e-mail-based surveys [8, 9]. Such response rate highlights the widely accepted challenge in addressing cartilage defects.

Debridement and MF were the treatments employed by most surgeons and most surgeons indicate to treat the medial femoral condyle with a single lesion. This is in accordance with the incidence of cartilage defects as reported from large epidemiologic database studies [10–14]. For symptomatic ICRS I/II defects, no surgical intervention, debridement and to a lesser extend MF were most considered as treatment when correspondents were asked. Debridement and MF were also most considered for ICRS III/IV defects up to 3 cm^2^_._ Similar trends were previously found in a German and Turkish survey [15, 16]. However, due to the mounting evidence of its ineffectiveness [17], both the 2011 and the 2019 DCS discouraged the use of MF in defects larger than 2 cm^2^. Hence, Dutch orthopedic surgeons seem to deviate from the DCS on this point.

In accordance with the DCS, the BMI limit for cartilage repair was set at 30 kg/m^2^ by 72% of the respondents. Although it has been well established that a BMI of 30 or larger is correlated with inferior outcomes after cartilage repair procedures [18], the prevalence of patients with such a BMI and the growth of this group is significant. [19].

Concomitant surgeries like meniscal repair and leg axis corrections were performed by a large majority of respondents. This is in accordance with recent German registry data [20]. Although the question related was asked from a symptomatic cartilage defect perspective, this survey did not scrutinize if cartilage repair could also have been concomitant to an anterior cruciate ligament (ACL) repair for instance. Such situation is conceivable when an unexpected cartilage defect is encountered during arthroscopy. The incidence of severe cartilage defects with ACL injuries for instance was previously found to be 16–46% [21]. On the other hand, there is also an increasing notion that combined treatments might decrease the risk for reoperation and improve outcomes [20, 22–24]. With a leg axis corrections considered at 5°–6° malalignment, most correspondents follow the DCS. Similar cut-offs were found in a previous West European study [16]. Some experts in the field, however, have advocated to correct malalignment in the mechanical axis from 2 degrees or more to unload the treated compartment and enhance repair. [22, 25] In a recent German database study the cut-off for varus axis correction was 3° [23]. Emphasized by the obesity pandemic, it is of great relevance to further clarify the indication and cut-off values for alignment corrections or overcorrections since unloading is potentially beneficial for the repair [22, 26]. In addition, the large spread in the cut-off values indicated by the respondents further confirms that there is yet no consensus for when to perform a corrective osteotomy in cartilage repair.

In general, respondents indicated a low employment of more complex techniques such as ACI, MF augmentations, allografts and FKRIs. At the same time, when specifically asked, these very treatments were indicated by the respondents for larger defects with higher ICRS scores, or, in older patients. This discrepancy suggests that there is a lack of availability of treatments. In addition, the previous debate in the Netherlands concerning the cost-effectiveness and hence reimbursement of ACI is possibly related to this [27]. Similar issues were also previously reported by Elmali et al. [15] Other explanations include the availability of allografts in Europe which is—contrary to the US—hampered due to regulatory issues [28]. But perhaps the best explanation for this is the centralization of cartilage repair in the Netherlands, highlighted by a 95% referral rate on indication. Indeed, due to regulation in the Netherlands, more complex techniques such as ACI or combined surgeries are only allowed to be performed in expertise centers. Hence, the orthopedic surgeon working in the periphery may only use debridement, MF and small osteochondral autografts.

Only half of the respondents would treat patellofemoral defects, which is a surprising finding given the fact that over 1/3 of patients present with patellofemoral defects [29]. Perhaps surgeons are discouraged by the inferior outcomes in this compartment [30]. At the same time, respondents also indicated to treat tibial defects, which are considered expert level treatments with only limited evidence and often inferior outcomes [31]. Hence, the latest DCS discouraged the surgical treatment of tibial defects. In line with this is the 8% of respondents treating kissing lesions which is also discouraged. Perhaps the fact that 28% of respondents seeing patients with multiple or kissing lesions did not work in a tertiary center contributed to these unadvised treatment indications.

Loose cartilage bodies were only fixed by the majority of the respondents if there was residual bone present, i.e., osteochondral shells. This is a critically important finding since a recent study indicated that pure chondral loose bodies could in fact serve as a functional autograft, even without the need for anchoring biomaterials [32]. Moreover, the patients’ own cartilage could potentially also serve as chondrocyte or chondron source for ACI and the novel minced cartilage repair options [33].

### Patient age-related considerations

Cartilage defects have been shown to be a major risk factor for osteoarthritis (OA) [3, 4]. One of the great challenges in the orthopedic community is to prevent or delay the onset of knee (early-) OA and thus prevent or delay total knee arthroplasty (TKA) [3]. Particularly, middle-aged patients—i.e., undergoing TKA in their 50s—have a high risk for revision surgery later in life [34]. Unfortunately, the fastest growing age-group undergoing cartilage repair or TKAs are the middle-aged patients [3]. Postponing TKA by means of long-lasting cartilage repair has therefore become a pressing topic. Not coincidentally, the International Cartilage Repair Society changed their society name by including ‘joint preservation’ in 2018.

The middle-aged patient is underrepresented in most of the studies investigating cartilage repair [3, 35]. It is not surprising therefore that in present survey there was a smaller degree of agreement in the results of respondents choosing treatments for older patients. Roughly 60% of respondents would consider MF as treatment in patients over 40 years of age. Importantly, when asked, most respondents did not see being middle-aged as a negative variable in cartilage repair. In fact, almost none (0-3%) of the respondents indicated that they believed any of the treatments to be highly affected by advancing age. Previous studies, however, have shown a negative effect by age on MF outcomes [3], and the detrimental effects of failed MF on consecutive treatments [3]. A recent systematic review concluded that more complex therapies such as cell-based therapies (ACI, bone marrow aspirate therapies), allografts or FKRIs have greater potential in older individuals [3].

With the aging population, it is also becoming increasingly important to evaluate the outcome of various cartilage repair treatments for different patient age categories. A major drawback in such age categorized research is that chronological age and biological age are obviously not the same. Biomarkers which differentiate in joint homeostasis are critically needed as they potentially can determine the ‘joint age’ rather than only relying on chronological age [36]. Combining biomarker data with a non-biased international registries could aid in understanding the prognostic factors of each treatment on individual level and age.

## Limitations

The major limitation of present study are its subjective outcomes, which is inherent to the nature of a survey study [37]. In the absence of CPT codes to register individual cases and different repair techniques, we are unable to compare current results to objective epidemiologic values. The results of the present study should therefore be interpreted as Dutch orthopedic surgeons describing how they would treat a given patient and defect, not as a completely objective measure of how to they actually treat their patients. Nevertheless, with the assumption of relatively similar demographics, the results of present studies can be compared to large database studies [11–14] and other survey studies [15, 16]. A nationwide registry system, analogous to or combined with the Dutch Arthroplasty Register, for different cartilage defects and prognostic factors could provide objective data, rather than relying merely on subjective data. 

Since we restricted the inclusion to members of orthopedic knee associations we only included surgeons with affinity for knee surgery. The very low volume orthopedic surgeon operating in a small peripheral hospital may therefore not be included. However, only a small number of respondents indicated to work in an expertise center and the results of the non-experts could consequently be extrapolated to the general orthopedic surgeon. Perhaps the knowledge gaps in present study would be even more profound in those who are not a member of a knee association.

## Conclusion

In the absence of a nationwide cartilage repair registry, this survey gives an impression of cartilage repair in the Netherlands. The present survey study showed that cartilage defects are treated by experts and many by non-experts. Both groups revealed a relative adherence to (inter)national guidelines. Small (< 2 cm^2^) and simple cartilage defects in the absence of additional injuries or malalignment may therefore be treated by general orthopedic surgeons if they follow the latest national recommendations. However, several knowledge gaps for specific defect and patient characteristics were shown, indicating that not everyone is fully aware of the latest insights. Caution should be exercised concerning the opportunistic use of MF, treating rare defects such as defects > 2 cm^2^ or those in the presence of loose viable cartilage bodies. Particularly patients with suboptimal characteristics such as an increased age (> 40 years), high BMI or malalignment should be considered for referral. This survey indicated that the recently introduced centralization of cartilage care is widely adopted in the Netherlands, which potentially aids in better knowhow and availability of advanced treatments, consequently better outcomes, and perhaps, joint preservation. Future research should focus more on dominant demographics such as older patients with typical comorbidities. This study should encourage orthopedic surgeons to engage in (inter-) national cartilage registries. Combining these registries with the Dutch Arthroplasty Register could aid in understanding the conversions to arthroplasties. Structural support from both the government and industry is necessary to enable the proper registration of all cartilage surgeries and products.

## Data Availability

Data from this study can be made available by authors upon request.

## Appendix 1: Survey questions

### General

1. You are:
  - Orthopedic surgeon
  - Resident
2. I use the following techniques for cartilage repair of symptomatic cartilage defects: (multiple answers possible)
  - Debridement/nettoyage
  - Microfracture
  - Autologous Chondrocyte Implantation (all generations)
  - Microfracture augmentations (such as Autologous Matrix-Induced Chondrogenesis [AMIC])
  - Osteochondral Allografts
  - Mosaicplasty (Osteochondral Autograft Transfer System [OATS])
  - Non-degradable cartilage implants (Metal/Polymer)
  - Biodegradable osteochondral scaffolds (such as Trufit™, MaioRegen, Agili-C™)
3. My experience with the treatment of cartilage defects is:
  - None
  - 0–1 year
  - 1–5 year
  -  > 5 years
4. Approximately how many cartilage defects have you treated in the past year? (open)
5. In general I treat cartilage defects within the following age categories: (multiple answers possible)
  -  < 18 years
  - 18–30 years
  - 30–40 years
  - 40–60 years
  - > 60 years
6. I carry out cartilage repair in patients who smoke:
  - Yes
  - No
7. When treating cartilage defects, I apply an upper limit for BMI (kg/m^2^) of:
  -  ≤ 25
  -  < 30
  -  < 35
  -  < 40
  - No limit
8. I apply cartilage repair to the following compartments of the knee: (multiple answers possible)
  - Medial femoral condyle
  - Lateral femoral condyle
  - Trochlea
  - Patella
  - Tibia plateau
9. Within the same knee I would address: (multiple answers possible)
  - Single lesions
  - Multiple lesions
  - Kissing lesions
10. In patients with a symptomatic cartilage defect I apply the following techniques in addition to cartilage repair: (multiple answers possible)
  - Surgical correction leg axis (tibiofemoral and patellofemoral)
  - Ligamental correction (cruciate, medial patellofemoral ligament, collaterals)
  - Meniscus surgery (nettoyage, sutures, allograft implants)
  - Other (please elaborate)
11. Starting from how many degrees (varus/valgus) would you carry out a surgical correction of the leg axis?: (open)
12. I refer patients to one of the specialized centers:
  - Yes
  - No
  - I work in such a center myself

### Specific

13. I would treat symptomatic, ICRS grade 1/2, cartilage defects with a maximum size of, with the following techniques: (multiple answers possible)

**Table:**

	< 1 cm^2^	1–2 cm^2^	2–3 cm^2^	3–4 cm^2^	> 4 cm^2^	Not
Debridement/nettoyage						
Microfracture						
Autologous chondrocyte implantation (all generations)						
Microfracture augmentations [(such as autologous matrix-induced chondrogenesis (AMIC)]						
Osteochondral allografts						
Mosaicplasty [osteochondral autograft transfer system (OATS)]						
Non-degradable cartilage implants (metal/polymer)						
biodegradable osteochondral scaffolds (e.g., Trufit™, MaioRegen, Agili-C™)						

14. I would treat symptomatic, ICRS grade 3/4, cartilage defects with a maximum size of, with the following techniques: (multiple answers possible)

**Table:**

	< 1 cm^2^	1–2 cm^2^	2–3 cm^2^	3–4 cm^2^	> 4 cm^2^	Not
Debridement/nettoyage						
Microfracture						
Autologous chondrocyte implantation (all generations)						
Microfracture augmentations [such as autologous matrix-induced chondrogenesis (AMIC)]						
Osteochondral allografts						
mosaicplasty [osteochondral autograft transfer system (OATS)]						
Non-degradable cartilage implants (metal/polymer)						
Biodegradable osteochondral scaffolds (as Trufit™, MaioRegen, Agili-C™)						

15. I would treat deep cartilage defects myself (ICRS grade 5/deeper than 6.5 mm):
  - Yes
  - No
16. In case of a cartilage corpus liberum I attempt refixation: (multiple answers possible)
  - No
  - Yes in case of an osteochondral corpus liberum
  - Yes in case of a chondral corpus liberum
17. I would consider the following techniques for the following age categories, irrespective of ICRS grade): (multiple answers possible)

**Table:**

	< 10 years of age	10–20 years of age	20–30 years of age	30–40 years of age	40–50 years of age	60–70 years of age	> 70 years of age
Debridement/nettoyage							
Microfracture							
Autologous Chondrocyte Implantation (all generations)							
Microfracture augmentations [such as autologous matrix-induced chondrogenesis (AMIC)]							
Osteochondral Allografts							
Mosaicplasty [osteochondral autograft transfer system (OATS)]							
Non-degradable cartilage implants (Metal/Polymer)							
Biodegradable osteochondral scaffolds (such as Trufit™, MaioRegen, Agili-C™)							

18. I consider the effect of middle age (40–65 year) on the success rate on the following techniques to be:

**Table:**

	Low		Average		High
	1	2	3	4	5
Debridement/nettoyage					
Microfracture					
Autologous chondrocyte implantation (all generations)					
Microfracture augmentations [such as autologous matrix-induced chondrogenesis (AMIC)]					
Osteochondral Allografts					
Mosaicplasty [osteochondral autograft transfer system (OATS)]					
Non-degradable cartilage implants (metal/polymer)					
Biodegradable osteochondral scaffolds (such as Trufit™, MaioRegen, Agili-C™)					

19. My clinic has a specific cartilage repair rehabilitation protocol
  - No
  - Yes, please elaborate (brace, loading etc.)
